# Evaluating computing students' digital skills and health literacy: A case from Bulgaria

**DOI:** 10.3389/fpubh.2022.1085842

**Published:** 2023-01-24

**Authors:** Kalinka Kaloyanova, Nikoleta Leventi, Elitsa Kaloyanova

**Affiliations:** ^1^Faculty of Mathematics and Informatics, Sofia University St. Kliment Ohridski, Sofia, Bulgaria; ^2^Faculty of Public Health, Medical University—Sofia, Sofia, Bulgaria; ^3^Department of Data Science and Content Creation, 365 Data Science OOD, Sofia, Bulgaria

**Keywords:** health literacy (HL), digital health literacy, Health Literacy Questionnaire (HLQ), digital competences, digital competences frameworks, DigComp 2.2, university students

## Abstract

The main purpose of this article is to describe the importance and the challenges of digital health literacy as recognized during the COVID-19 pandemic. First, basic definitions of health literacy and digital health literacy are provided, followed by, and matched against digital competence frameworks, and health literacy skills content and scales. Based on that, a compatibility analysis is provided, against the expectations for satisfactory levels definition for the respective competences and skills. For the approbation of the approach, results received from the participation of computing students at the Sofia University St. Kliment Ohridski in the COVID-19 Health Literacy Survey are used.

## 1. Introduction

The concept of literacy has changed significantly in recent years, and today, it is increasingly associated with the acquisition of specific skills in certain areas. Literacy has an impact not only on the professional career but also on the overall lifestyle of people, especially when it comes to health literacy. On the contrary, the evolution in the way information is presented and perceived today, related to its storage and processing in electronic form, brings to the forefront the need for solid digital skills.

These trends were reinforced during the COVID crisis when the need to access reliable information was vital, and most of this information was only available digitally.

### 1.1. Digital literacy

The demand for digital skills for life and work today is greater than ever. Broadly considered as skills needed to effectively operate in an increasingly digital world, digital skills are analyzed at different levels, and many frameworks discuss their evaluation.

First, several industry-based competence frameworks were explored in the IT area. Between them, the Skills Framework for the Information Age (SFIA) and the European e-Competence Framework (e-CF) are the most widely used.

SFIA addresses the Professional Skills, Behaviors, Knowledge, Qualifications, and Certifications of the employees. Seven generic “Levels of Responsibility”—Follow, Assist, Apply, Enable, Ensure/advise, Initiate/influence, and Set strategy/inspire/mobilize, are defined to measure them, and specific skills are defined for each of the seven levels (1).

The European e-Competence Framework (e-CF), a reference framework of information and communication technology (ICT) competences, structures the required competences in this field into five e-Competence areas, namely, Plan, Build, Run, Enable, and Manage, following the classical IT lifecycle view. This is the first (Dimension 1) of the four dimensions proposed by the framework. Dimension 2 gives a general description of the competences, while Dimension 3 describes five e-CF proficiency levels for each competence. Finally, Dimension 4 presents knowledge and skills examples, which are related to those described in Dimension 2 competences (2).

As most jobs (not only in IT) require digital skills today, in 2013 the European Commission (EC) launched a common framework, The Digital Competence Framework (DigComp), to assist in the evaluation of these skills among European citizens. The DigComp Conceptual Reference model differentiates digital competences into five competence areas as follows (3).

1. Information and data literacy.
2. Communication and collaboration.
3. Digital content creation.
4. Safety.
5. Problem-solving.

For all twenty-one competences in the five competency areas (CA), proficiency levels are described in categories as *Foundation, Intermediate, Advanced*, and *Highly professional*.

The most recent version, DigComp 2.2, complements the previous ones with connections to emerging technologies, as well as to other organizations. In addition, it provides more guidelines and examples (4).

### 1.2. COVID-19 as a pandemic

COVID-19 is an infectious disease caused by the SARS-CoV-2 virus (5). In March 2020, the World Health Organization (WHO) described the spread of COVID-19 as a pandemic (6). One of the first things that were explained in order to slow down the transmission and prevent ourselves was for people to be very well-informed about the disease symptoms and how the virus spreads. Now, in the third year of the COVID-19 pandemic, information about the virus spreads on the Internet. Meanwhile, misinformation is still a problem that we have to deal with.

According to the WHO, nowadays societies confront a health decision-making paradox (7). The paradox is the necessity for people to make healthy lifestyle choices for themselves and their families, while being neither prepared nor supported in their efforts to make the right choices. The result is that today's advanced societies are still unprepared to equip people with the required skills in order to find, understand, assess, and use the provided information to improve their health.

It was this paradox that played a crucial role during the COVID-19 pandemic. It was then realized that the weaker the health literacy skills, the less healthy the choices people make.

### 1.3. Health and digital health literacy

There are many definitions for health literacy (HL). One of the first definitions, well-accepted, is the one proposed in 2012 by the European Health Literacy Consortium: “Health literacy is linked to literacy and entails people's knowledge, motivation and competences to access, understand, appraise, and apply health information in order to make judgments and take decisions in everyday life concerning health care, disease prevention, and health promotion to maintain or improve quality of life during the life course” (8).

Different studies have been conducted analyzing the importance of HL, exploring different geographies, and covering various population samples and student groups (9, 10). Some studies focus on and further evaluate the impact of the COVID-19 pandemic on students' behavior and health promotion (11, 12).

Many tools are developed that measure health literacy. According to the latest health literacy research and practices described and developed by the Ophelia (OPtimising HEalth LIteracy and Access) process (13), we have now several tools that allow us to identify and respond to health literacy needs (14).

- Health Education Impact Questionnaire (heiQ) (15): It is used to evaluate health education and self-management programs.
- Information and Support for Health Actions Questionnaire (ISHA-Q) (16): It is used to identify specific health literacy strengths and limitations, it can be used for individuals and communities, and it was designed for cultures that often make decisions as a group;
- eHealth Literacy Questionnaire (eHLQ) (17): It provides insight into users' perceptions and experiences of digital health solutions, and it helps understand why implementations work or fail.

One of the most popular tools used in health literacy measures in the world is the Health Literacy Questionnaire (HLQ), which enables needs assessment, evaluation, and quality improvement (18, 19). The major characteristic of HLQ is that it helps to determine a person's ability to obtain, read, understand, remember, and act on healthcare information. It gives insight into health literacy strengths and limitations and helps us to develop suitable interventions. It assesses nine literacy areas and can be used for individuals and communities.

In practice, HLQ is a multidimensional tool for measuring health literacy, and this makes it convenient for the purpose of our study. It has nine scales, and each scale measures an aspect of health literacy as follows.

#1. Feeling understood and supported by healthcare providers.
#2. Having sufficient information to manage my health.
#3. Actively managing my health.
#4. Social support for health.
#5. Appraisal of health information.
#6. Ability to actively engage with healthcare providers.
#7. Navigating the healthcare system.
#8. Ability to find good health information.
#9. Understand health information well-enough to know what to do.

### 1.4. Purpose of the study

Achieving a good level of health literacy, more specifically e-health literacy, is an important factor for young people's prosperity in the modern world. The investigation of the current state in this new field requires purposeful efforts of both components, namely, digital competences and health literacy skills. This process should start at the universities and find its initial reflection in their curricula.

Many internationally respected and reputable organizations, including the Organization for Economic Co-operation and Development (OECD), have underlined the role and importance of HL. Addressing HL barriers will help health systems become more people-centered. Now health literate individuals are able to seek ways to understand their health options and take more control over their health decisions (20).

In this study, the following new questions arise:

- What are the digital competences required for the students studying medicine and vice versa?
- What are the health literacy skills of computing students?
- What needs to be improved, to make them work better together in the field of eHealth in their further professional realization?

Despite intensive research in this area during the last few years, as described above, there are still not enough specific tools to perform this assessment effectively. Another possible approach is to analyze results from already done research, where other goals and scope are set, and to look for elements that allow assessment of certain aspects of digital competences and health literacy skills and their matching.

Such a possibility was provided by the research done on the COVID-19 Health Literacy Survey: University Students (COVID-HL Survey) where an assessment of “digital health literacy of university students during the COVID-19 pandemic” is included (21). We explored such results from a COVID-HL Survey, provided among computing students in Bulgaria—namely, students in the Faculty of Mathematics and Informatics (FMI) at Sofia University St. Kliment Ohridski. The purpose of our analysis was to make an initial assessment of the level of health literacy of students in Bulgarian universities based on COVID-HL Survey results that include items related to students' health literacy and digital skills.

## 2. Materials and methods

In this study, we use the results received from the COVID-19 Health Literacy Survey on the digital health literacy of university students during the COVID-19 pandemic, conducted with the participation of students at the Faculty of Mathematics and Informatics at Sofia University. We match those results against the Digital Competence Framework (DigComp 2.2) and the health literacy measurement tool Health Literacy Questionnaire (HLQ). Our primary objective is to provide an analysis of compatibility, against the expectations for satisfactory levels definition for the respective competences and skills.

### 2.1. The survey

The COVID-19 Health Literacy Survey was developed as a tool to assess some aspects of the “digital health literacy of university students during the COVID-19 pandemic” (21). The tool was developed by both the Public Health Center Fulda (PHZF) at the Fulda University of Applied Sciences and the Interdisciplinary Center for Health Literacy Research at Bielefeld University. The questionnaire has been used for exploring the behavior of students in many countries during information seeking and has been proven to be a reliable tool (22, 23).

Twenty-eight questions in the following four groups were included in the survey.

- Sociodemographic information (Q1–Q10);
- Current life situation and future (Q11–Q12);
- Health literacy and information-seeking behavior (Q13–Q23);
- Personal health situation (Q24–Q28).

Each investigated element had several sub-components and a relevant scale for assessment.

The survey was translated into Bulgarian, and some details, concerning Bulgarian websites and Bulgarian institutions, were adjusted. The study was conducted at Sofia University St. Kliment Ohridski, the biggest and the oldest university in Bulgaria (24, 25). The Faculty of Mathematics and Informatics (FMI) at Sofia University has strong traditions in conducting high-level education in the fields of Mathematics, Informatics, and Computer Science. More than 80% of undergraduate students at the faculty are educated in Computer Science, Information Systems, and Software Engineering programs based on the latest ACM curricula recommendations (26).

In total, 1,690 computing students from the Faculty of Mathematics and Informatics were asked to take part in the survey, and 221 students participated in the study. The students were informed that although some personal data were collected, it could not be assigned to a specific person. Furthermore, the information was collected solely for scientific purposes, with the aim of additionally developing support services. The survey had the approval of the ethics committee of Sofia University St. Kliment Ohridski. All answers were collected electronically *via* a digital platform ensuring the anonymity of the participants. Of all respondents, 84% were undergraduate students in Computer Science, Software Engineering, and Information Systems programs at FMI; 15% were graduate students; and 1% were Ph.D. students. In the biggest group of undergraduate students, 32.62% were from the first academic year, 22.99% were from the second academic year, 24.60% were from the third academic year, and 19.79% were from the fourth academic year.

The study explores the HL of a relatively homogeneous group of computing students. Some limitations of the data collected are also done by the use of the predefined COVID-19 Health Literacy Survey. Additionally, compliance with the frameworks chosen for the study imposes limitations on the volume of data used. On the contrary, it makes the research more focused and provides insights, which we hope can further help in better understanding students' HL and finding ways of improving in the field.

### 2.2. Digital competences scale: DigComp 2.2

Different frameworks discuss among others digital competences and e-competences. We decided to use DigComp 2.2 because it provides the latest and more integrated view of the topic. Considering the digital literacy aspects in our study, we focus on the first competence area in the framework: 1. Information and data literacy. Three competences are presented as follows.

1.1 Browsing, searching, and filtering data, information, and digital content.
1.2 Evaluating data, information, and digital content.
1.3 Managing data, information, and digital content.

All these three competences are connected to the ability to search, navigate, and access data, and to evaluate the reliability of data sources and the information provided by them. The same skills are the focus of one of the main goals of the COVID-HL Survey questionnaire—“assess digital health literacy of university students during the COVID-19 pandemic” (21), and several questions from the questionnaire address the topic.

The other competence area where we find a match within the terms of the discussed frameworks is the fourth competence area in the DigComp 2.2 list: 4. Safety (4). The competence area 4.3. Protecting health and wellbeing concerns the ability of people to avoid health-related risks and threats to their physical and psychological wellbeing while they use digital technologies.

### 2.3. Health literacy scale: HLQ

The HLQ provides nine independent scale scores, focusing on the strengths and limitations of the respondent and providing insight into those scales. Populations' health literacy strengths and limitations can be evaluated by average scale scores for groups of respondents (along with standard deviations). The effect sizes before and after a concrete intervention can be evaluated through the differences in the mean scores either before or after the intervention of different groups. Finally, similar health literacy profiles of groups of individuals can be evaluated by using cluster analysis. As “HLQ was grounded in citizens' lived experience” (19), it is expected to be useful in the assessment of citizens' needs, which corresponds to the DigComp 2.2 framework.

In our case, we selected four out of the nine scales: #5. Appraisal of health information, #7. Navigating the healthcare system, #8. Ability to find good health information, and #9. Understanding health information well-enough to know what to do. Based on them, we conducted a compatibility analysis of the received COVID-HL Survey questionnaire results, by an evaluation regarding the low and high levels of the implied attributes defined in HLQ.

The selection of four out of nine HLQ scales was done on the basis of the similarity of the scales in all the HL survey (21) data, the HLQ (19), and the DigComp 2.2 framework (4). Further data should be collected to cover the rest of the HLQ scales and develop a complete tool.

### 2.4. Frameworks matching and cross-reference

Having the above-discussed frameworks and scales, we analyzed the content of all items and selected the questions from the COVID-19 Health Literacy Survey that most closely correspond to both frameworks (DigComp 2.2 and HLQ). We focused our conceptual analysis on:

- identifying common elements in both frameworks—DigComp 2.2 and HLQ and their recommendations and questionnaire questions that address them; and
- analyzing the available responses against the framework of the digital competences and addressing the health literacy scales.

The result of the matching is presented in Table 1.

**Table 1:** DigComp 2.2, HLQ, and COVID-HL-Survey cross-reference.

**DigComp 2.2 competence area**	**DigComp 2.2 competence description**	**COVID-19 health survey questions**	**HLQ scales**
1. Information and data literacy	1.1 Browsing, searching and filtering data, information, and digital content	Q14	#8. Ability to find good health information
	1.2 Evaluating data, information, and digital content	Q16, Q22, Q23	#5. Appraisal of health information
	1.3 Managing data, information, and digital content	Q19, Q20	#7. Navigating the healthcare system
4. Safety	4.3 Protecting health and wellbeing	Q11, Q12	#9. Understanding health information well-enough to know what to do

We concentrated our survey on items directly covered by corresponding questions from the COVID-19 Health Literacy Survey and as such do not use all items from both frameworks (DigComp 2.2 and HLQ).

In the next sections, we present the selected questions, discuss the received responses, and analyze them in accordance with the above guidelines.

## 3. Results

For the purpose of this study, we reviewed the available questions from the COVID-19 Health Literacy Questionnaire and their responses. According to DigComp 2.2, Dimension 1 defines several competence areas, and Dimension 2 defines the competences for each area. We match the respective competence area and the competences listed within it to a question or multiple questions from the COVID-19 Health Literacy Survey as well as to the HLQ health literacy areas whenever we find a correspondence between the three components, as stated in Table 1.

### 3.1. Information browsing, searching, and filtering as an ability to find good health information

Under the scale of *(**1**)* *Very easy*, *(**2**)* *Easy*, *(**3**)* *Difficult*, and *(**4**)* *Very difficult*, three sub-questions.

Q14-op.1… make a choice from all the information you find?
Q14-op.2… use the proper words or search query to find the information you are looking for?
Q14-op.3… find the exact information you are looking for?

were provided for question Q14 “When you search the Internet for information on the coronavirus or related topics, how easy or difficult is it for you to…”

**Figure 1:**
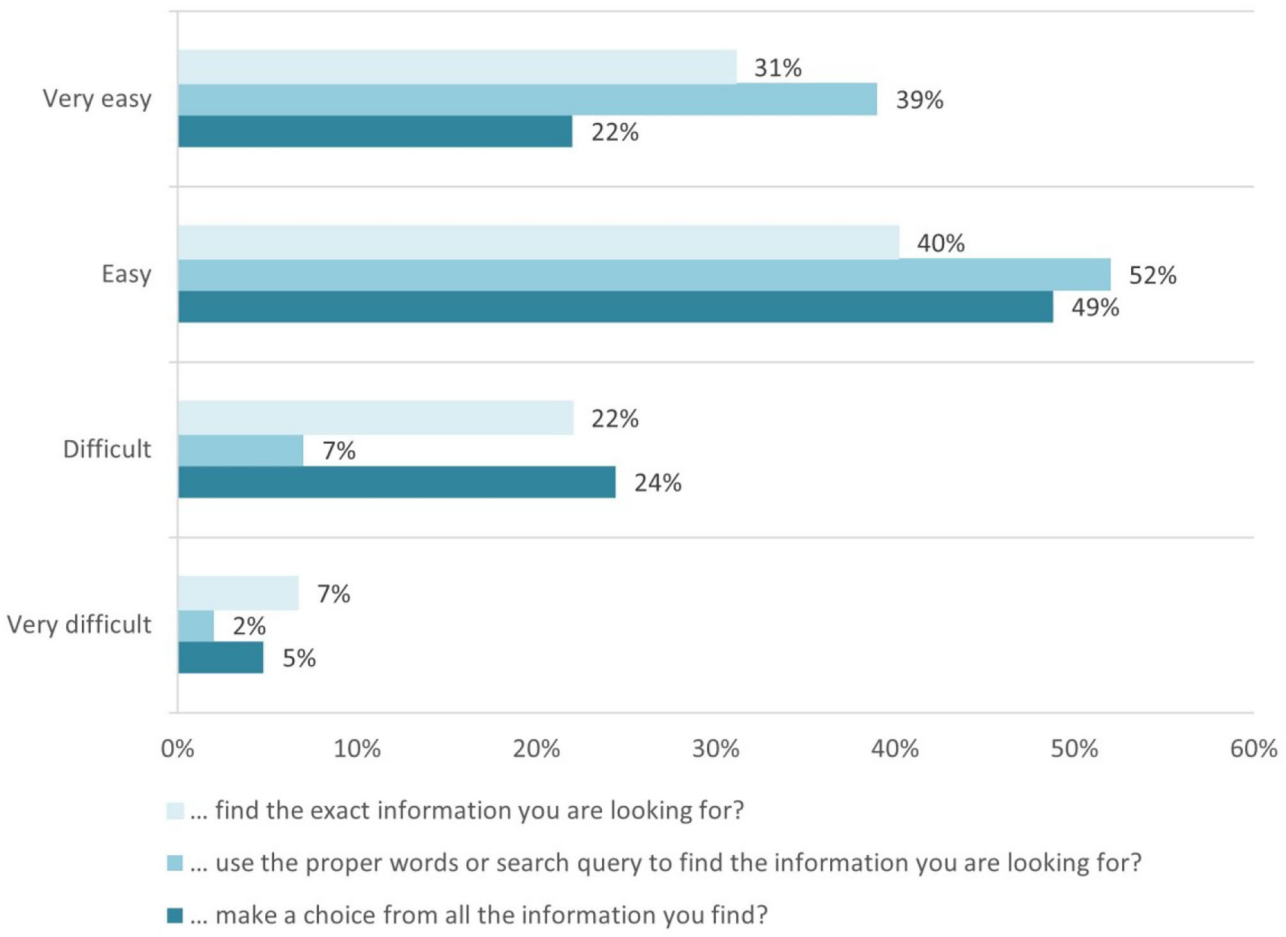
Evaluation of students' ability easily to find good health information.

In total, 71% of students reported *Easy* and *Very easy* they find the information they are looking for, and 91% of students consider it *Easy* and *Very easy* to use the proper words for a focused search. Also, most of the students find it *Easy* and *Very easy* to make a choice from the information they find (see Figure 1).

This declares skills covered in the competence area 1. Information and data literacy, more particularly for the competence 1.1 Browsing, searching and filtering data, information, and digital content, aiming “To articulate information needs, to search for data, information, and content in digital environments, to access them and to navigate between them” (4).

From the health literacy side, such skills are outlined in scale #8. Ability to find good health information on HLQ health literacy areas. The low level of the construct is described as “Cannot access health information when required. Is dependent on others to offer information,” while the high descriptor of the construct is defined as “Is an information explorer. Actively uses a diverse range of sources to find information and is up to date.” According to the definitive answers to question Q14, we can conclude that the highest indicator has been achieved.

### 3.2. Evaluation of data, information, and digital content as an appraisal of health information

To evaluate competence 1.2. Evaluating data, information, and digital content of competence area 1. Information and data literacy, we use three questions from the COVID-19 Health Literacy Survey—Q16, Q22, and Q23. We match the results to the HLQ scale #5. Appraisal of health information.

We started the analysis with the question Q16 “When you search the Internet for information on the coronavirus or related topics, how easy or difficult is it for you to…” with the following three sub-questions.

Q16-op.1… decide whether the information is reliable or not?
Q16-op.2… decide whether the information is written with commercial interests (e.g., by people trying to sell a product)?
Q16-op.3… check different websites to see whether they provide the same information?

**Figure 2:**
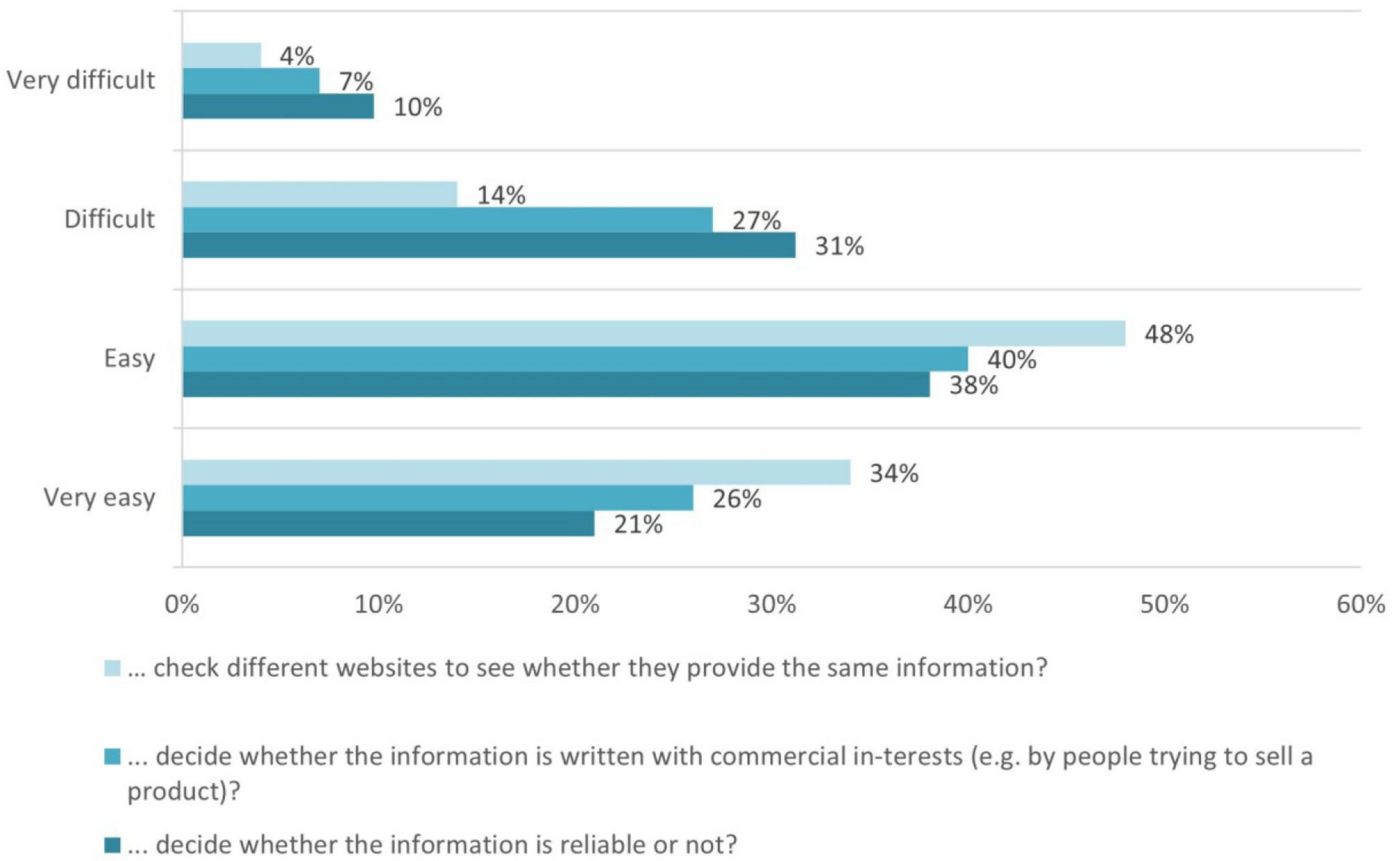
Means the students used to appraise health information.

The results present relatively lower levels for appraising the reliability of the information: 31% of respondents find it *Difficult*, 10% of respondents find it *Very difficult* to decide whether the information is reliable, and 59% of respondents find it *Easy* or *Very easy* to apply their critical thinking skills and to make this decision. It is not quite easy for the students to evaluate whether the information is written with commercial interests-−34% of respondents consider it Difficult and *Very difficult* (Figure 2).

As for the option of checking different sources, the results show that 48% of the students find it *Easy*, and another 34% of the students find it *Very easy*, to check different websites and compare whether they provide the same information.

Computing students' digital competences are also achieved by analyzing their answers to questions related to the evaluation of the reached information (Q22) and the satisfaction level achieved (Q23).

In the first question, Q22 “Now it's about how important various things are to you when you search the Internet for coronavirus and related topics. How important is it to you that…,” six sub-questions are provided as follows.

Q22-op.1 … the information is up to date?
Q22-op.2 … the information is verified?
Q22-op.3 … you quickly learn the most important things?
Q22-op.4 … the information comes from official sources?
Q22-op.5 … different opinions are represented?
Q22-op.6 … the subject is dealt with comprehensively?

**Figure 3:**
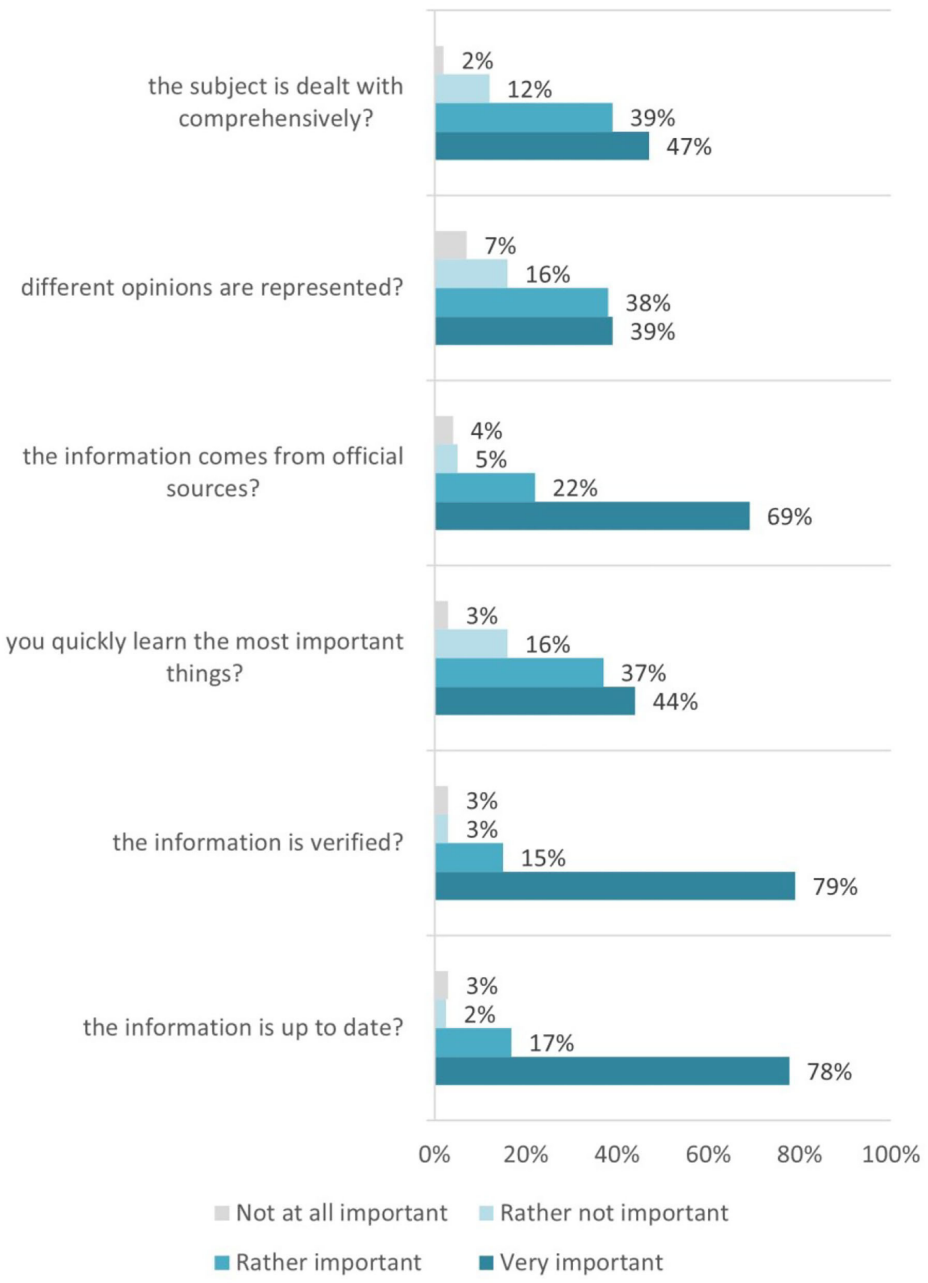
Reached information evaluation/appraisal criteria used.

The majority of the responses relay the importance of information being verified, secure (coming from official sources), and up-to-date for students. Such responses indicate that there are objective criteria for the health-specific information evaluation, and the computing students fully comply with this (Figure 3).

The last question relevant to data, information, and digital content evaluation—Q23 “How satisfied are you with the information you find on the Internet about coronavirus?”—measures students' satisfaction with the obtained information. The answers are provided under the scale of *(**1**)* *Very satisfied*, *(**2**)* *Satisfied*, *(**3**)* *Partly satisfied*, *(**4**)* *Dissatisfied*, and *(**5**)* *Very dissatisfied*.

**Figure 4:**
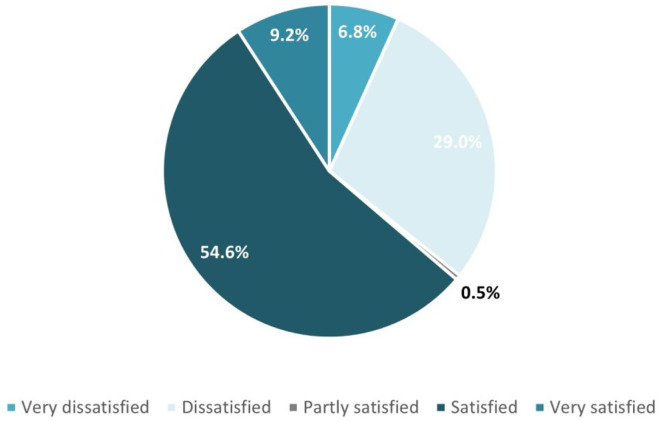
Reached information satisfaction levels achieved.

Generally, the answers report students successfully find the information they are looking for while critically evaluating the obtained information (Figure 4). This fully corresponds to the *Advanced* proficiency level of 1.2. Evaluating data, information, and digital content competence.

The overall results show that computing students reach also the upper levels of the high descriptor of the HLQ construct for area #5 defined as “Able to identify good information and reliable sources of information. They can resolve conflicting information by themselves or with help from others.”

### 3.3. Managing data, information, and digital content for navigating the healthcare system

Explored are the results from answers to the next two questions Q19 and Q20, in relation to competence 1.3. Managing data, information, and digital content (under the same first DigComp 2.2 competence area) and corresponding to area #7 of the HLQ—Navigating the healthcare system.

In question Q19, various possibilities are mentioned on how to get information about the coronavirus and related topics on the Internet.

Q19-op.1–Search engines (e.g., Google, Bing, Yahoo!).
Q19-op.2–Websites of public bodies (for example, the Bulgarian national unified information portal, the current news provided by the Ministry of Health, RHI—the Bulgarian Regional Health Inspectorate).
Q19-op.3–Wikipedia and other online encyclopedias.
Q19-op.4–Social media (e.g., Facebook, Instagram, and Twitter).
Q19-op.5–YouTube.
Q19-op.6–Blogs on health topics.
Q19-op.7–Guidebook-communities (e.g., zdrave.net).
Q19-op.8–Health portals (e.g., credoweb.bg).
Q19-op.9–Websites of doctors or health insurance companies.
Q19-op.10–News portals (e.g., of newspapers and TV stations).

**Figure 5:**
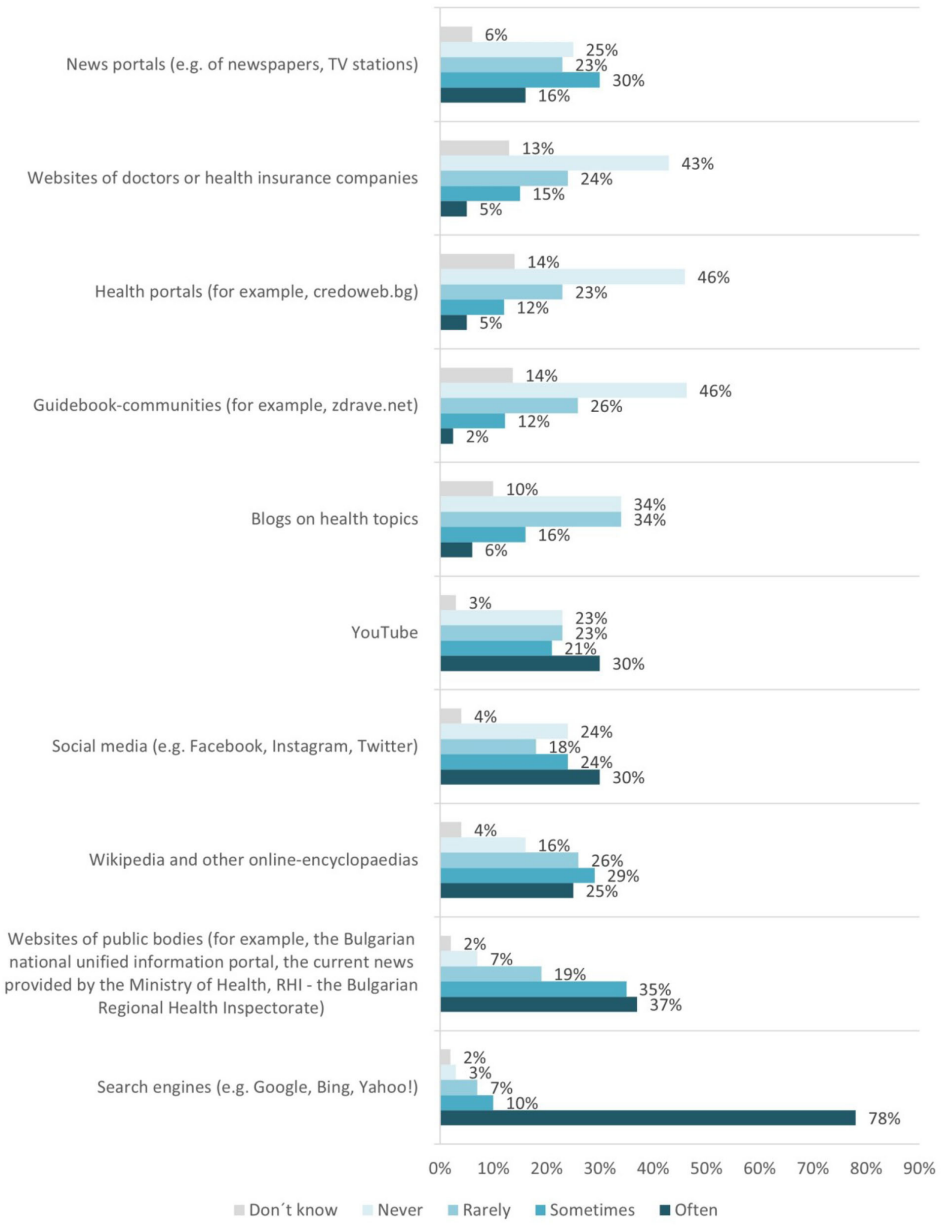
Sources used by the students to get information about the coronavirus and related topics on the Internet.

The results received for Q19 are interesting. The students need to indicate how often they use different sources to get information about the coronavirus. The list includes the most used search engines (like Google, Bing, and Yahoo) and specific sources of health information like websites of public bodies. Furthermore, specific Bulgarian websites, which provide up-to-day coronavirus information, were included in our adapted questionnaire (Figure 5).

There is a prominent trend showing that computing students trust reliable public sources and have reservations when it comes to trusting individual entities working or having paid interests in the field.

**Figure 6:**
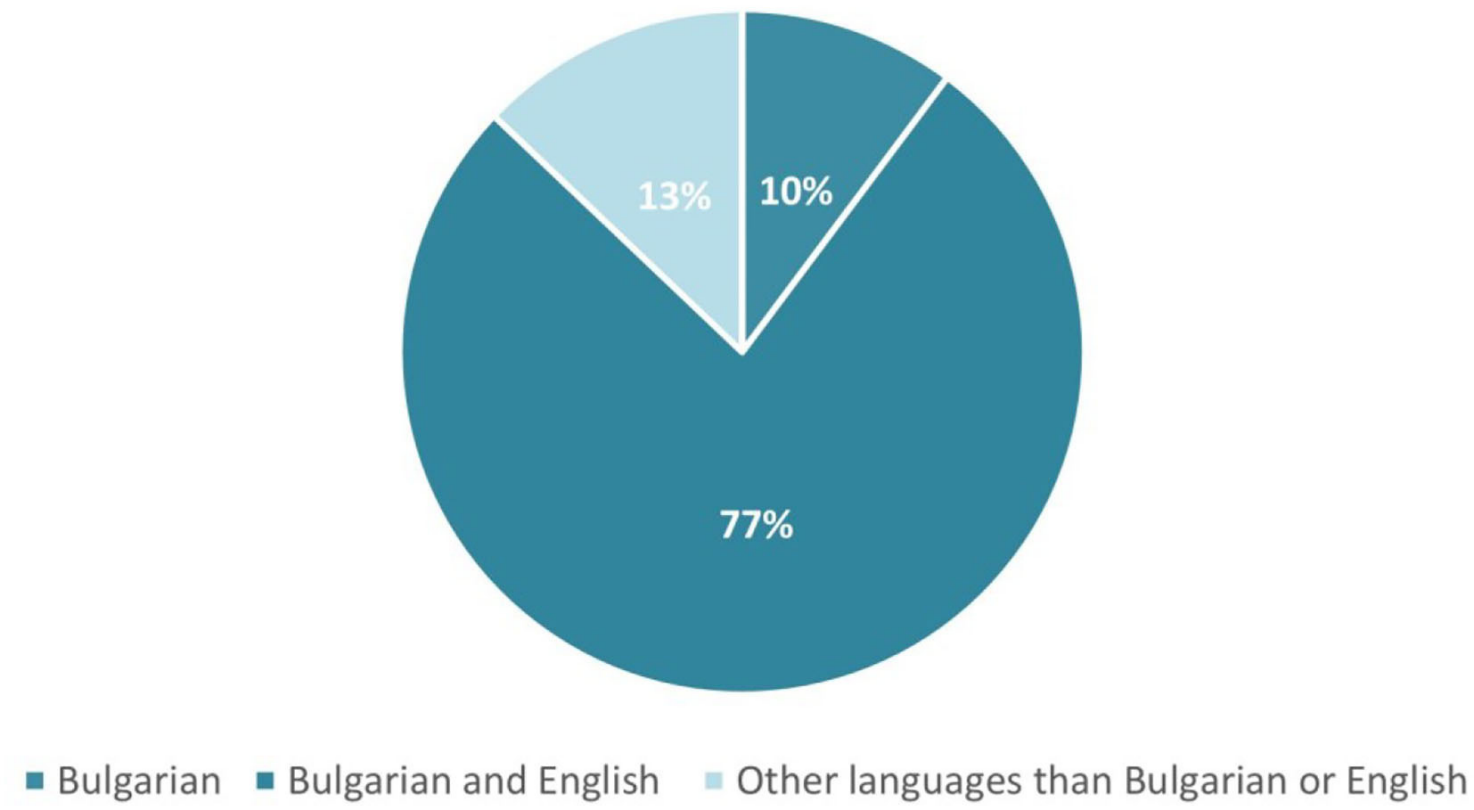
Language/s of the sources used for searching information on coronavirus and related health topics by the students.

For question Q20 “What language do the sources have that you use for searching information on coronavirus and related health topics?” we see English language preference strongly expressed, as 77% of respondents use it (Figure 6).

We believe that the provided results for Q19 and Q20 put the computing students to the upper level of the HLQ area #7 “Navigating the healthcare system,” taking into consideration the high-level construct for this area is defined as “Able to find out about services and supports so they get all their needs met. Able to advocate on their own behalf at the system and service level” (15).

### 3.4. Safety and protection of health and wellbeing as a measure for understanding health information well-enough to know what to do

Finally, for the evaluation of competence area 4. Safety, and particularly competence 4.3. Protecting health and wellbeing, we analyzed both questions Q11 and Q12 of the COVID-19 Health Literacy Survey.

**Figure 7:**
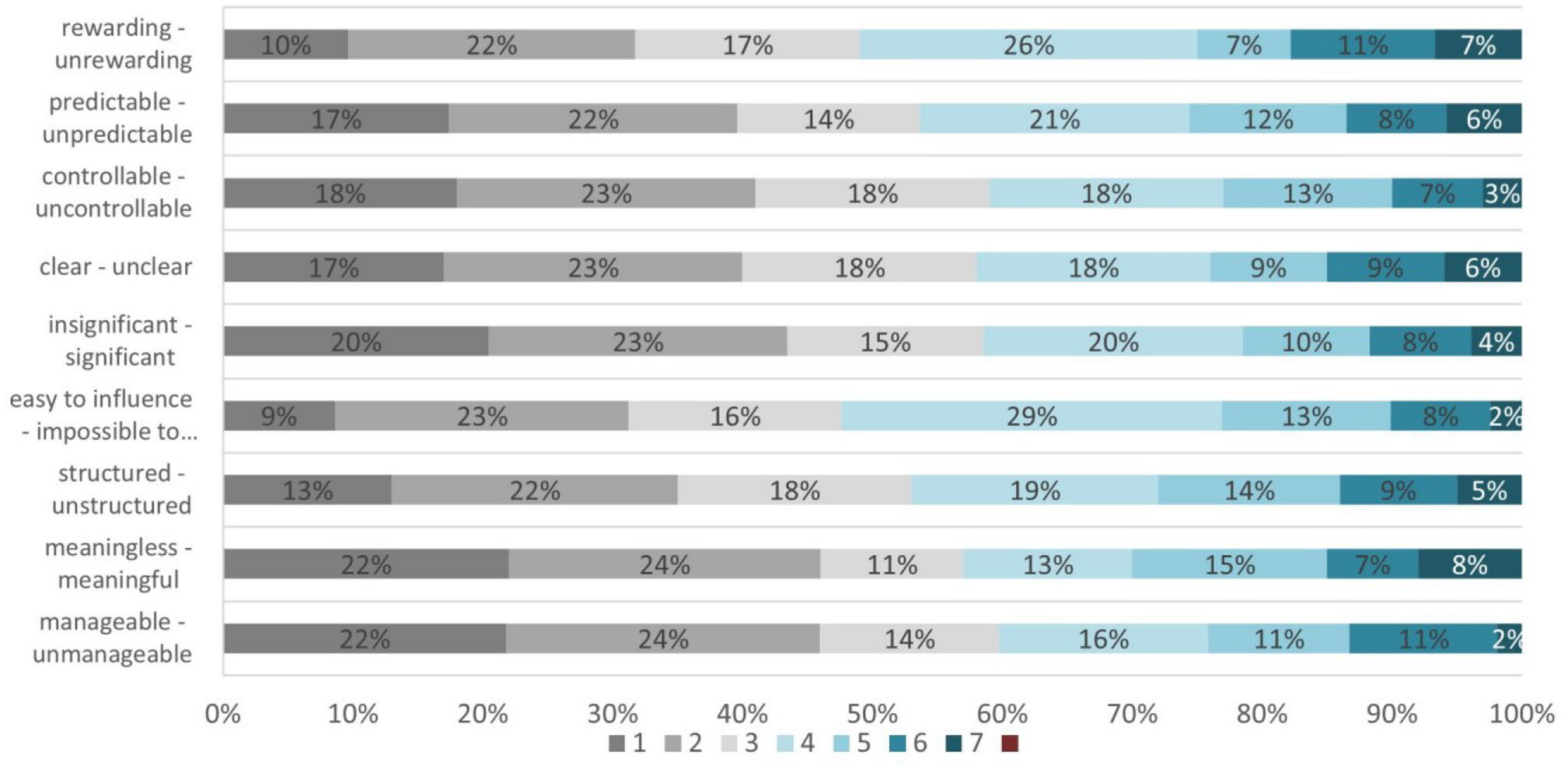
Students' wellbeing self-evaluation.

Question Q11 “How do you personally find your current life situation in general?” explores students' perception of eight options: (op.1) manageable–unmanageable, (op.2) meaningless–meaningful, (op.3) structured–unstructured, (op.4) easy to influence–impossible to influence, (op.5) insignificant–significant, (op.6) clear–unclear, (op.7) controllable–uncontrollable, (op.8) predictable–unpredictable, and (op.9) rewarding–unrewarding. A 7-level scale evaluates the nuances between the two opposite values (Figure 7).

The question focus is on measuring the sense of coherence mainly on the work context. This can be interpreted as a wellbeing evaluation criteria presence. This is the first component of the HLQ area #9 “Understanding health information well-enough to know what to do.” Three directions can be identified in these evaluations as follows: comprehensibility (options 1, 3, 6, and 9), manageability (options 4 and 7), and meaningfulness (options 2, 5, and 8). The overall results indicate that our students, having in mind the difficulties faced around the COVID-19 pandemic, are normally conservative against the whole situation.

Question Q12 provides statements that concern students' attitudes toward the future. Each statement can reflect their attitude to different degrees. If a certain statement describes the attitude exactly, it is answered with “decidedly true.” If the statement is not an accurate description of the attitude, it is answered with “decidedly false.” Otherwise, it is answered with “Hard to say.” Nine statements—options are provided as follows.

Q12-op.1–I am afraid that the problems which trouble me now will continue for a long time.
Q12-op.2–I am terrified by the thought that I might sometimes face life's crises or difficulties.
Q12-op.3–I am afraid that in the future my life will change for the worse.
Q12-op.4–I am afraid that changes in the economic and political situation will threaten my future.
Q12-op.5–I am disturbed by the thought that in the future I won't be able to realize my goals.
Q12-op.6–I fall into a state of tension and uneasiness when I think of my future affairs.
Q12-op.7–I am sure that in the future I will realize the most important goals (values) in my life.
Q12-op.8–I have the impression that the world tends toward collapse.
Q12-op.9–I am disturbed by the possibility of a sudden accident or serious illness (e.g., cancer, COVID-19).

The statements again are provided under a scale of seven degrees, this time from *(**1**)* *Decidedly true, to* *(**7**)* *Decidedly false*, with the middle option *[Hard to say]*.

The use of the future anxiety levels evaluation is divided into two sub-components for the short and the long anxiety versions. The aim is to compare any existing tendencies related to thinking about the future with anxiety. The existing uncertainty and any further disaster anticipation in the future can be used as an evaluation criterion for computing students' way of dealing with area #9 “Understanding health information well-enough to know what to do” of the HLQ areas.

We matched the results from questions Q11 and Q12 with area 9. “Understanding health information well-enough to know what to do” of the HLQ areas. The lower level construct is defined as “Has problems understanding any written health information or instructions about treatments or medications. Unable to read or write well-enough to complete medical forms.” The upper level of the construct is defined as “Is able to understand all written information (including numerical information) in relation to their health and able to write appropriately on forms where required.”

**Figure 8:**
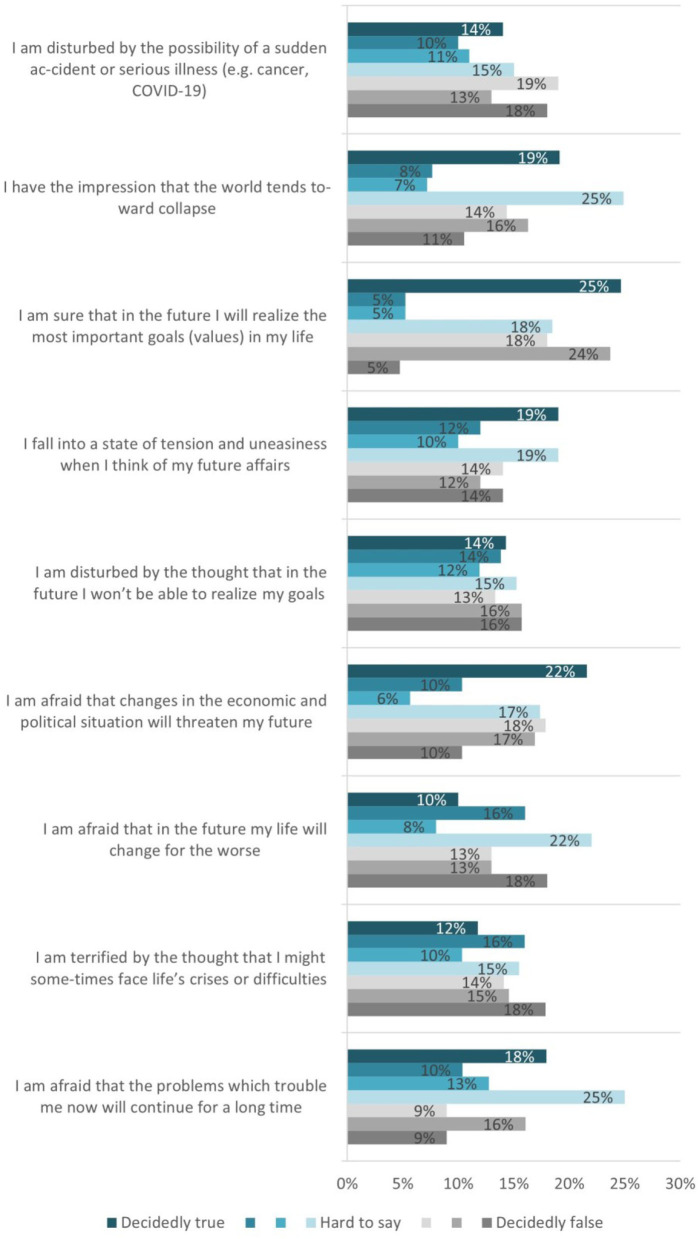
Students' health and wellbeing risk factors self-evaluation.

The results received show that although the computing students are well-oriented in searching, allocating, and evaluating health information, they do not feel confident about the future, and they do not have a clear view and knowledge of what to do in the future with the health information they have (Figure 8).

## 4. Discussion

This section goes further in reviewing the available questions from the COVID-19 Health Literacy Questionnaire and the computing students' responses. Following the conceptual qualitative analysis on identifying common elements in both frameworks, DigComp 2.2 and HLQ in the previous section, now a quantitative analysis is the focus.

The method used starts with applying the correlation analysis of the received responses per question, as a major tool. This analysis allows us to identify whether there is a relationship between certain variables and then helps to determine the magnitude of such a relationship. The second step is to evaluate the received responses and determine the proficiency level (Dimension 3 of DigComp 2.2) corresponding to the related competence (Dimension 2 of DigComp 2.2) for the competence area under evaluation (Dimension 1 of DigComp 2.2), recognized in the previous section.

### 4.1. Information browsing, searching, and filtering as an ability to find good health information

The correlation analysis conducted on the results of question Q14 “When you search the Internet for information on the coronavirus or related topics, how easy or difficult is it for you to …” indicates a strong linear relationship between the provided options. The levels of the association are very high (0.82, 0.92, 0.93), which means the students' responses are changing in the same direction.

Computing students' proficiency level regarding competence 1.1. “Browsing, searching and filtering data, information, and digital content” of DigComp 2.2 competence area 1. Information and data literacy is evaluated at the *Advanced* level. According to the responses received, computing students cover the requirements of the ability to assess information needs, adapt searching strategy, and explain how to access data.

### 4.2. Evaluation of data, information, and digital content as an appraisal of health information

The proficiency level for the next competence 1.2. Evaluating data, information, and digital content was calculated based on three questions from the COVID-19 Health Literacy Survey—Q16, Q22, and Q23.

Correlation analysis was conducted on the results for questions Q16 and Q22. For both questions, we have results with a strong linear relationship between the provided options. The levels of the association are very high, and the lowest coefficient of all is 0.69. This exposes an aligned approach toward students' responses. These results are also confirmed by the answers in Q23 “Reached information satisfaction levels achieved” (see Figure 4), where we have more than two-thirds of the students feeling satisfied or very satisfied (Satisfied 55%, Very satisfied 9%) with the information they find on the Internet about coronavirus.

All those indicate an *Advanced* proficiency level for competence 1.2. Evaluating data, information, and digital content of DigComp 2.2 competence area 1. Information and data literacy. Computing students can critically assess the credibility of sources, data, information, and digital content they find on the Internet about coronavirus.

### 4.3. Managing data, information, and digital content for navigating the healthcare system

Two questions, Q19 and Q20, were used in relation to proficiency level evaluation for competence 1.3. Managing data, information, and digital content under the first competence area of DigComp 2.2. A correlation analysis was conducted only for question Q19 (see Table 2), regarding the various possibilities of sources used to get information about the coronavirus and related topics on the Internet.

**Table 2:** Q19 “Sources used by the students to get information about the coronavirus and related topics on the Internet” correlation analysis.

	**op.1**	**op.2**	**op.3**	**op.4**	**op.5**	**op.6**	**op.7**	**op.8**	**op.9**	**op.10**
Q19-op.1	1.00									
Q19-op.2	0.67	1.00								
Q19-op.3	0.35	0.85	1.00							
Q19-op.4	0.60	0.77	0.80	1.00						
Q19-op.5	0.60	0.71	0.82	0.96	1.00					
Q19-op.6	−0.58	−0.36	0.18	0.09	0.22	1.00				
Q19-op.7	−0.61	−0.59	−0.16	−0.02	0.03	0.89	1.00			
Q19-op.8	−0.55	−0.57	−0.16	0.03	0.07	0.85	1.00	1.00		
Q19-op.9	−0.58	−0.53	−0.10	0.06	0.09	0.88	1.00	1.00	1.00	
Q19-op.10	−0.17	0.48	0.80	0.65	0.60	0.54	0.33	0.33	0.39	1.00

The correlation analysis results show that the different sources used can be grouped into categories varying from extremely strong positive relationships down to strong negative relationships. That means we have options with strong positive linear relationships toward each other, as well as options with a strong negative relationship, for example, students who trust, and often (op.2) use websites of public bodies (e.g., the Bulgarian national unified information portal, the current news provided by the Ministry of Health, and RHI—the Bulgarian Regional Health Inspectorate); and tend to distrust and not use (op.6) Blogs on health topics, (op.7) Guidebook-communities (e.g., zdrave.net), (op.8) Health portals (e.g., credoweb.bg), or (op.9) Websites of doctors or health insurance companies. At the same time, those students trust (op.4) Social media (e.g., Facebook, Instagram, Twitter) and (op.5) YouTube, despite the risks related to the use of unchecked sources of medical information.

This lack of clear indications for navigating the healthcare system is confirmed by the results of question Q20, related to the language/s used for searching for information on coronavirus and related health topics the sources discussed. That means that computing students have the ability to also rely on English to cross-check the acquired information.

The proficiency level for competence 1.3. Managing data, information, and digital content of the first DigComp 2.2 competence area is evaluated as *Intermediate*. Computing students cannot go above selecting data, information, and content and organizing them. No signs of abilities related to manipulating such data are provided.

### 4.4. Safety and protection of health and wellbeing as a measure for understanding health information well-enough to know what to do

The proficiency level for Competence 4.3. Protecting health and wellbeing of DigComp 2.2 competence area 4. Safety calculation was done based on questions from the COVID-19 Health Literacy Survey—Q11 and Q12. Correlation analysis was conducted for both questions.

The correlation analysis conducted on the results of the question Q11 “How do you personally find your current life situation in general?” indicates in general very strong linear relationship between the provided options, excluding two options, which can be evaluated as not well-understood, as they do not change the overall evaluations. The levels of the association are in general very high, which means the students' responses are consistently changing in the same direction.

The future anxiety levels analyzed by the correlation analysis results on question Q12, where student present their attitude to the future, reveal that answers to the different statements vary from strong and extremely strong positive relationships down to negative relationships. We have options changing in value, in the opposite direction than other options (Table 3).

**Table 3:** Q12 “Students' health and wellbeing risk factors self-evaluation,” correlation analysis.

	**op.1**	**op.2**	**op.3**	**op.4**	**op.5**	**op.6**	**op.7**	**op.8**	**op.9**
Q12-op.1	1.00								
Q12-op.2	−0.16	1.00							
Q12-op.3	0.29	0.85	1.00						
Q12-op.4	0.48	−0.02	0.12	1.00					
Q12-op.5	0.32	0.72	0.65	0.43	1.00				
Q12-op.6	0.63	0.16	0.43	0.76	0.44	1.00			
Q12-op.7	0.57	−0.24	−0.08	0.92	0.35	0.56	1.00		
Q12-op.8	0.85	0.05	0.41	0.81	0.51	0.83	0.79	1.00	
Q12-op.9	−0.13	0.34	0.32	0.37	0.33	0.37	0.22	0.33	1.00

There are no clearly identifiable trends regarding the health and risk factors evaluation. The computing students do not seem to understand health information to such a degree as to know what to do.

The proficiency level for competence 4.3. “Protecting health and wellbeing” of DigComp 2.2 competence area 4. Safety is evaluated as *Foundation*. Computing students can only differentiate, select, and identify ways to avoid health risks and threats.

## 5. Conclusion

Today, different competence frameworks and scales for assessing literacy in different domains exist. Particularly important are those related to health literacy, or rather digital health literacy and related digital skills. In this article, we presented the use of two tools, namely, DigComp 2.2 and HLQ, to assess the health literacy and digital skills of Sofia University computing students.

Although a targeted full study was not done and results from a previous study were used, which has some relevance to the digital competences and literacy levels discussed, the obtained results are promising. We conclude that the students show good coverage of the levels specified in these two widely used frameworks—they cover almost completely the formulated quality standards on several major indicators of both frameworks. The study found a stable level of health literacy in FMI students in several health literacy scales, although some cases reported not properly understanding health information to know what to do. These good results are mainly due to the high level of digital skills of the student of the Faculty of Mathematics and Informatics.

These findings confirm what WHO calls the health decision-making paradox, and reveal that the improvement in digital health literacy should be closely linked to the achievement of a good level of common digital skills, particularly in information and data literacy. The application of computer-based knowledge and skills to specific organizational context, like health area, could also be important for a future professional career and should be developed over time with both education and expertise. Most educational programs in the Faculty of Mathematics and Informatics support curricula that purposefully shape the good digital skills of students, which helps them maintain a good level of literacy in various fields. At the same time, in order to overcome any of the shortcomings identified, a clearer mention of the aspects of identified areas should be addressed in the next revision of the programs.

## Data availability statement

The raw data supporting the conclusions of this article will be made available by the authors, without undue reservation.

## Author contributions

All authors listed have made a substantial, direct, and intellectual contribution to the work and approved it for publication.
